# Medical Department of the Army

**Published:** 1859-07

**Authors:** 


*Medical Department of the Army.*—The Army Medical Board, which recently convened in this city, have recommended the following gentlemen for appointment in the Medical Staff of the Army:—

No. 1. George Suckley, M. D., New York.

No. 2. Dewitt C. Peters, M. D., New York.

No. 3. Charles H. Alden, M. D., Pennsylvania.

Assistant Surgeons, Alexander B. Hasson and Jonathan Letherman, were examined by the board, and found qualified for promotion.

